# A Tangible Programming Tool for Children to Cultivate Computational Thinking

**DOI:** 10.1155/2014/428080

**Published:** 2014-02-25

**Authors:** Danli Wang, Tingting Wang, Zhen Liu

**Affiliations:** ^1^Beijing Key Lab of Human-Computer Interaction, Institute of Software, Chinese Academy of Sciences, Beijing 100190, China; ^2^Chongqing Medicine Exchange Co., Ltd., Chongqing 404100, China

## Abstract

Game and creation are activities which have good potential for computational thinking skills. In this paper we present T-Maze, an economical tangible programming tool for children aged 5–9 to build computer programs in maze games by placing wooden blocks. Through the use of computer vision technology, T-Maze provides a live programming interface with real-time graphical and voice feedback. We conducted a user study with 7 children using T-Maze to play two levels of maze-escape games and create their own mazes. The results show that T-Maze is not only easy to use, but also has the potential to help children cultivate computational thinking like abstraction, problem decomposition, and creativity.

## 1. Introduction

Computational thinking (CT) is a term coined by Wing [1] to describe a set of thinking skills, habits, and approaches that are integral to solving complex problems using a computer and widely applicable in the information society. There is growing consensus [2] that computational thinking is a fundamental skill that everyone needs to succeed in our complex and technological culture. CT makes it possible for children to improve the analytical ability that may be helpful in both STEM (science, technology, engineering, and mathematics) subjects and many other professional areas, even in daily life [3]. The advantage of this early exposure to CT is that it will help children to build a solid foundation of algorithmic and data structures—the basic nuts and bolts of the mechanics of computer programming [4]. Computer programming is an excellent way to develop computational thinking skills [5]. Grover takes programming as a useful way for kids to learn problem solving and computational thinking [3]. Research has indicated that learning how to program computers can have a positive and measurable effect on children's achievement, not only in areas such as math and science, but also in language skills, creativity, and social emotional interaction [6, 7]. However, the fact remains that programming for children is still just plain hard and requires strong motivation on the child's part in order to succeed [5]. Thus, the programming environment must strongly motivate the subject by placing it in a context that is so compelling and meaningful to the child that he/she does not give up.

Tangible programming is to make programming an activity that is accessible to the hands and minds of young children by making it more direct and less abstract. Tangible programming may have an appeal even to experienced abstract thinkers [8]. By combining computer programming and tangible interaction, tangible programming allows children to manipulate “codes” directly, which makes programming more appealing. Besides, using physical objects to interact with computer is easier to involve children in the process [9]. Research involving tangible interaction and children has often focused on how tangibles might support or improve learning compared to more traditional methods [10, 11]. This research is mainly about the capability of tangible programming to help children with some CT skills. The physical manipulation acts like a scaffold between real world and virtual world, which may contribute to the abstraction in CT. We assume that tangible programming is also an efficient approach to teach children about CT.

In this paper, we propose T-Maze, a tangible programming environment designed to allow children to build computer programs by manipulating a set of wooden blocks which are interconnected by magnets. We conduct a study involving 7 participants (aged 5–9) using T-Maze to play maze-escape games and create their own mazes to explore how CT concepts take shape for children in a tangible programming environment. The results may provide a lens through which one can consider the implications for learning and teaching computational thinking.

## 2. Background and Related Work

### 2.1. Computational Thinking (CT)

Computational thinking is closely related to, if not the same as, the original notions of procedural thinking developed by Sey in *Mindstorms* [12]. In a 2006 article, Wing discussed computational thinking as “a way of solving problems, designing systems, and understanding human behavior that draws on concepts fundamental to computer science” [1]. To successfully broaden the awareness of computer science, efforts must be made to lay the foundations of CT in an early stage of children's development [13]. The impact of CT on disciplines covers philosophy, physics, education, and so forth [2] and CT concepts have been used as a basic element for several efforts aimed at more precise, deeper, and wider interpretation of computing [14].

Research regarding the implementation of computational thinking skills in informal education provides valuable insights. This includes attention to K-12 curriculum, general education at colleges and universities, and interdisciplinary research [15–17]. The National Academies' Computer Science and Telecommunications Board held a series of workshops on “Computational Thinking for Everyone” with a focus on identifying the fundamental concepts of computer science that can be taught to K-12 students. The first workshop report [18] provides multiple perspectives on computational thinking. The International Working Group on Computational Thinking [19] points to several successful projects that use simulation and modeling, robotics, and computer game design to teach abstraction, automation, and analysis. As they note, these kinds of activities also involve an iterative design, refinement, and reflection process that is central to creative as well as computational thinking [20].

Research involving graphical programming and CT nurturing has also gotten some progress. Researchers used Alice as a tool to support the development of algorithmic thinking, problem solving, and event processing [4, 21, 22]. It is shown that Alice does help children in the development of abstract thinking, result analyzing, and automation understanding [22]. And Scratch was chosen as an appropriate platform for teaching student CT through musical coding [23]. The visual feedback that students get from Alice and Scratch allows them to relate the program to the action they see on the screen and helps them refine their programs [4]. The promising performance of graphical programming in children's computational ability development serves as a good spur for us to look into similar potential of tangible programming.

CT involves defining, understanding, and solving problems, reasoning at multiple levels of abstraction, understanding and applying automation, and analyzing the appropriateness of the abstractions made [22]. However, there is yet no consensus on how to assess these skills [18]. Therefore, in this paper, particular attention is paid on the potential capability of a tangible programming tool to help children cultivate some CT skills like abstraction, automation, problem decomposition, and analysis. We describe the results in regard to our research questions, but we do not draw conclusions about learning outcomes with regard to computational thinking, instead, we try to get to know about the computational concepts in children's programming activity.

### 2.2. Tangible Programming

Since the 1960s a large number of programming languages targeted at novice users have been created [24]. Novice computer programming systems aim to ease or eliminate the process of learning language syntax. Beyond syntax, there are many specific conceptual hurdles faced by novice programmers [25]. A relatively recent approach to ease the learning process has been the creation of tangible programming languages [9].

Electronic Blocks [26] are physical Lego blocks designed to allow young children (aged 3–8) to create Lego forms with interesting behaviors. It consists of three types of building blocks: sensor block as input; logic block to compute; behavior block as output. Each block is embedded with a processor or other essential electronic devices, which dramatically increase the cost of each piece of the token and the entire system. Besides, the syntax is simple with relatively limited blocks available.

Tangible Programming Bricks [8] are Lego blocks that can be stacked together to form programs. Each block could accept a single card, allowing users to communicate with other blocks via IR transmission. By stacking blocks together with accompanying cards, users can teach toy cars to dance, kitchen users to program microwaves, and toy trains to react to signals. But the problem is that the way to manipulate the blocks is complicated for children and the programming is in a procedural style with a lack of advanced programming constructs like conditional and so forth.

Tern [11, 27] is a tangible programming language designed to allow children to use a collection of interlocking wooden blocks to build physical computer programs. Each block represents an action, a flow-of-control construct, a parameter, or a sensor value. The blocks are low cost, but children need to manually use a camera to capture the image of block sequence which is then transferred to computer to compile. Thus, without immediate feedbacks about the outcomes of the physical programs, real-time test and debug are not supported in Tern.

Toque [28] uses concrete real world cooking scenarios as programming metaphors to support an accessible programming learning experience. User can control the cooking process and simulate some cooking motions by body movements like stir-fry action and so forth. There is a distinct procedural workflow in this programming experience, but not sufficient for programming learning in practice.

TurTan [29] is a desktop tangible programming system designed for Turtle Geometry, which is based on LOGO. It uses a camera to capture and recognize the real-time position of fingers and objects on the desktop. However, compared with blocks, the interactive desktop is expensive and not easy to control for children.

Recently, there is a more generally defined tangible programming work from MIT called Twinkle: Programming with Color [30]. It uses a color sensor to read colors from physical objects, drawings, or collages. And those colors are mapped to certain outputs, like sounds, graphics, or robotic movements. It is remarkable that the objects can be anything at hands but the system seems not programming-rich.

From the above, we find that many programming tools are high cost because of the embedded electronic equipment (e.g., Electronic Blocks, Tangible Programming bricks), Wii and Interactive Desktop (e.g., Toque, TurTan). That makes them not easily available for children in developing countries. Tern is low cost and has rich programming concepts, but children need to manually take photos of the blocks to actually run the program, which is especially not appropriate for young children. To this end, we are devoted to provide a low-cost tangible programming environment appropriate for children. Also, as to computational thinking, there is little research about the computational skills nurturing capability of these programming tools.

In this paper, we present T-Maze for children (aged 5 to 9) to build programs by constructing physical blocks and introduce it more in terms of how computational thinking was considered in the tool. T-Maze uses wooden blocks to represent basic programming commands and computer vision technology to convert physical programs into digital code automatically. Sensors are integrated into the system to invite interaction and attempt to let children experience the event trigger mechanism.

## 3. Implementation of T-Maze

### 3.1. T-Maze Overview

**Figure 1:**
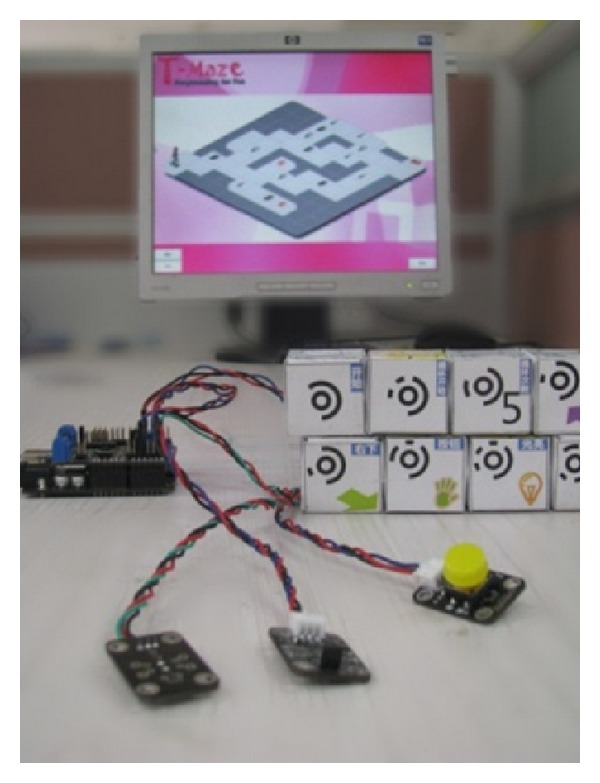
T-Maze overview: sensors, wooden blocks, camera, and software.

T-Maze [31] is a tangible programming tool for children (ages 5–9) to build programs to play multilevel maze-escape games and create their own mazes, by manipulating a collection of wooden blocks. In a maze-escape game, children connect wooden blocks to control a virtual avatar in a grid world on screen to escape from a maze. To create a new maze, children use the same set of blocks representing a whole maze map and the passable paths.  Figure 1 shows the main part of T-Maze.

### 3.2. Tangible Programming Blocks

**Figure 2:**
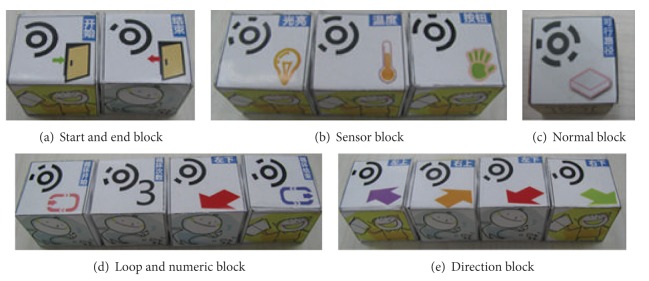
Tangible programming blocks.

Tangible programming blocks are inexpensive and durable cubes with no embedded electronics or power supplies. We hypothesized that the use of familiar objects (wooden cubes) would transform an unfamiliar and potentially intimidating activity like computer programming into an inviting and playful experience. There are five kinds of programming blocks (Figure 2) in T-Maze: start and end block, sensor block, normal block (passable path block used in maze creation), direction block, and loop and numeric block. For each block, two little button magnets are attached on each of the two opposite sides. The magnet attraction and repulsion can somehow tell children whether the block of now is placed in a correct angle or used with the correct side. The way that magnets interconnect the blocks in a straight line helps children arrange their program snippets and also is beneficial to computer vision processing.

For each type of blocks, the symbols are different. But generally, each symbol has three parts: computer vision identifier (the circular, black-and-white symbols printed on each block), simple descriptive texts, and an icon indicating the function.

### 3.3. Sensors

**Figure 3:**
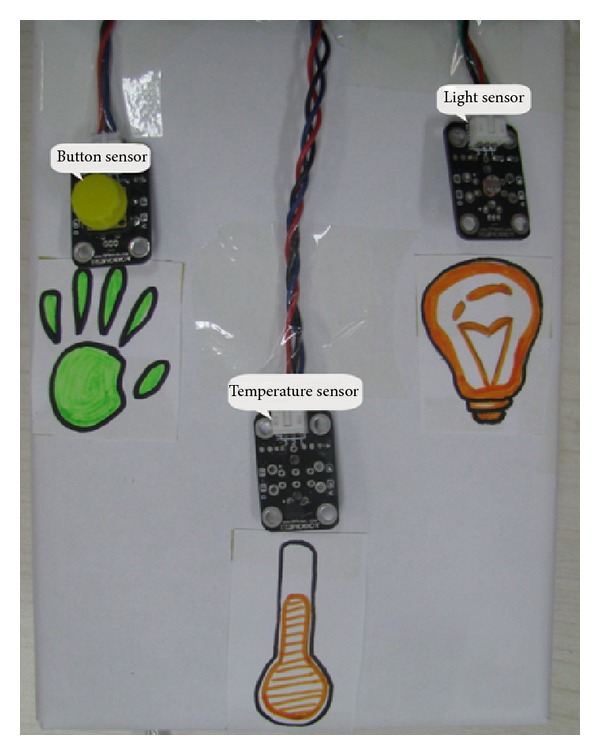
Physical sensors in T-Maze.

T-Maze has three sensors: temperature sensor, light senor, and button sensor (Figure 3). The three sensors are represented by three sensor blocks (Figure 2(c)), which generate three corresponding squares in virtual maze map on screen. In a program execution, when the avatar reaches one of these squares in the maze, the child must do something with the sensors (e.g., cover a light sensor) to allow the avatar to proceed. Sensors are introduced here to invite interaction between children and the system, meanwhile attempt to help children get familiar with event trigger mechanism in programming.

### 3.4. Computer Vision System

T-Maze uses TopCode [32] computer vision identifier (the circular, black-and-white symbols printed on each block in Figure 2) to convert physical programs into digital code automatically. The identifiers allow the computer vision system to identify each block and determine its position in relation to the other blocks. A digital camera with an image resolution set to 1280 by 960 pixels is attached to a laptop PC. A programming area approximately 40 cm wide by 30 cm high can be reliably compiled as long as the area is white or light-colored. Captured images are transferred to the host computer through a USB connection and saved as JPEG images on the file system. With this image, the compiler converts a program directly into virtual machine code.

### 3.5. Game Modules

T-Maze has two game modules: Maze Escape and Maze Creation. Game and creation are activities which have good potential for computational thinking skills [33]. Mazes are compelling and meaningful to most children for their experience in school or day-to-day life. The maze metaphor is used because we think that placing programming in this context could motivate the child so that he/she does not give up.

**Figure 4:**
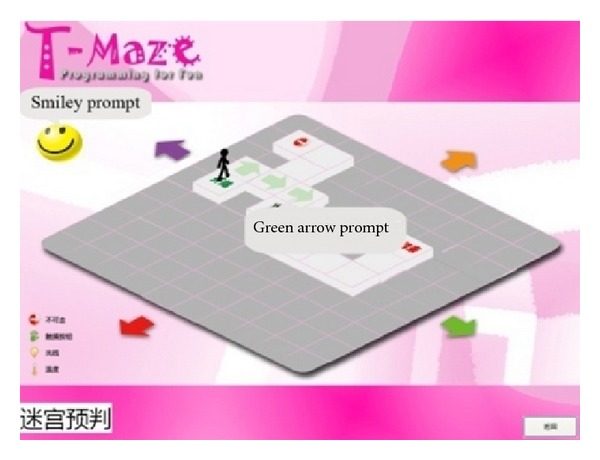
Interface of one level maze-escape game.

**Figure 5:**
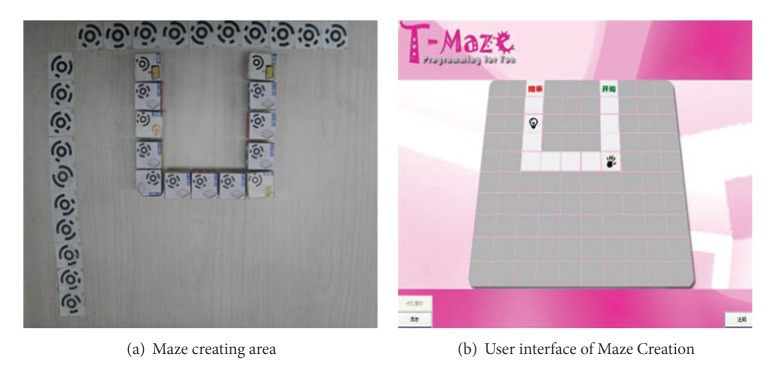
The creating area (a) and user interface (b) of Maze Creation.

Problem analysis takes place during the process of testing and debugging their programs [22]. The computer vision system captured images automatically enabling T-Maze to give real-time graphical and voice feedbacks, which help children with the analysis. For example, in Maze Escape (Figure 4), when a block is placed correctly, the background of the occupied square will change into a green arrow. Otherwise, the smiley on the upper left screen will turn upset along with a voice prompting the possible solutions like “maybe, I need a sensor” or “did I walk too many steps in this direction?” In Maze Creation (Figure 5), the real-time visual feedback tells children “what you see is what you get” and provides them a live creating experience.

## 4. CT in T-Maze Programming

CT is a comprehensive term that covers abstraction, automation, analysis, creativity, and so forth [14]. In this paper, we select to focus on several concepts that can be observed in T-Maze.

### 4.1. Abstraction

Abstraction ability is to find appropriate level of detail to define and solve a problem [34]. With T-Maze, children could simulate the abstract model of certain mazes in reality, map themselves into the virtual characters on screen, and control the characters' behaviors. For example, to play a maze-escape game in T-Maze, children need to build a path for their avatars using the physical blocks. In order to know how these blocks function, children need to think about how to perceive the world coordinates of the virtual maze and how to map the behaviors in real world, like move straight or make a left turn, into the virtual characters' behaviors in the programs. As to the sensors, there are three layers of abstraction-reality mapping, that is, sensor cells in virtual maze, physical sensor blocks, and physical sensors.

### 4.2. Automation

Automation is a labor saving process in which a computer is instructed to execute a set of repetitive tasks. It is much more efficiently compared to the processing power of a human and the automated execution of process by machine is going to change everything [35]. In this light, computer programs are “automations of abstractions.” The program children built in T-Maze automates the simulation of escaping from maze, using a run loop which updates the feedbacks on screen and interacts with users on the basis of designed rules for sensors.

### 4.3. Problem Decomposition and Analysis

Decomposition may take the form of stripping down a problem to what is believed to be its bare essentials [22]. Breaking problems down into smaller parts that may be more easily solved and analyzing to reuse them can enable and simplify the resolving of more complicated and larger-scale problems. And, analysis is a reflective practice which validates whether the abstractions are correct. In programming, they would analyze whether the avatars' behavior is expected and whether there are some conditions that are not taken into account during the abstraction phase. Children also engage in analysis when they judge whether their abstractions are efficient. This analysis may help them optimize and find a better solution to the problem.

In T-Maze, the entry of the maze is marked with green texts, and the exit is marked with red texts. Children are guided to get a blueprint of the path to be built from these markers. After knowing the ports where the path starts and ends, children have to connect them by “paving” the cells in mazes with programming blocks. During this process, children are encouraged to analyze and compare their plans and choose the one that leads to the shortest path. To this end, the feasible path in one maze is not unique. And several sections of the paths are designed to be straight in one direction, where the loop block may be a better choice. However, it is not worthy to use the loop block if the straight section of the path has less than four cells. Though this is a little ticklish for children, it is where the analysis is needed. Also, T-Maze gives real-time feedbacks, which help children with the analysis when testing and debugging their programs.

### 4.4. Creativity

Creativity is both a comprehensive capability of human beings and an important part of the CT skill set [14]. We try to develop children's creative skill by stimulating their curiosity, especially the creative imagination. Creation is at the root of creative thinking [20]. The tangible programming tool should enable children to process any abstracting, simulating, and creating activities freely. The freedom may open children's minds, a key point in promoting children's creativity.

And as such, the Maze Creation module is designed for this purpose, which offers children freedom to create any shape of mazes as they like. It is interesting to see what children would come up with from the creating.

## 5. User Study

We have conducted several studies to evaluate T-Maze in terms of its usability. These studies involved 20 children (aged 5–9) using T-Maze to program in a laboratory environment. In the latest study, we focused on the potential capability of T-Maze to nurture computational thinking in children's programming activities.

### 5.1. Research Design

#### 5.1.1. Research Questions

For this study, we were interested in several research questions with an emphasis on the last one.

1. Is T-Maze easy to use for children?
2. Do children like programming with T-Maze and what are they interested in?
3. How do the four CT concepts take shape for children in programming?

Though the first two questions have been examined in previous studies, we wanted to be sure that children are comfortable to involve in our study every time. Considering the diversity of users and usage scenarios, we regard the usability of T-Maze as a long-term test subject, especially when some new modifications or improvements need to be verified. Also, we expect usable feedbacks from children, if any, to help us improve the tool, since it is always preferable to have more children like T-Maze.

#### 5.1.2. Tasks

**Figure 6:**
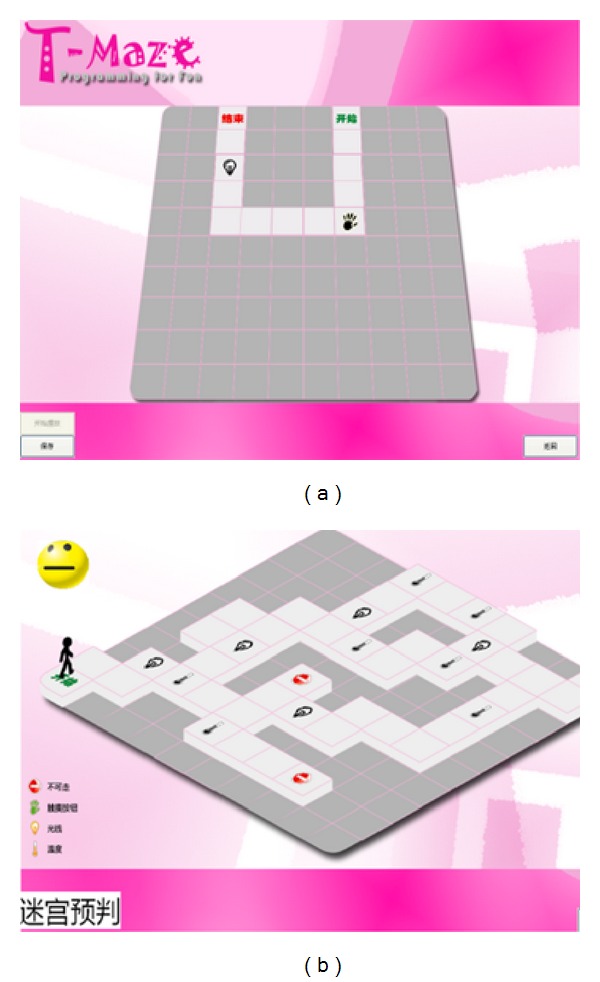
The simple (a) and complex (b) maze-escape games in the study.

We conducted two sessions in the study: maze escape and maze creation. In maze escape session, children were asked to complete two different levels of maze-escape games, which were designed in consideration of individual ability difference, as well as the occasions where problem decomposition and analysis probably happen. To play a maze-escape game, children had to find a passable path in the maze for the avatar to traverse through by building programs with wooden programming blocks. The simple maze-escape game only had one passable path and would need 9 blocks at most (without the use of Loop blocks), while the complex one had multiple passable paths and would need variable number of blocks according to the length of chosen path (Figure 6). For a maze creation task, children were given no restrictions to create any maze they liked by constructing new maze maps with the same set of blocks. There was no time limitation in both sessions. Throughout the study, we had a comfortable seat in front of the table for children to sit when they were working on the tasks, as well as a rest area for them to wait or rest.

#### 5.1.3. Interviews and Questions

For the interviews after the two main sessions, we wanted to know children's opinions about the tool, their programming experience, or anything that remained in their mind. So the questions asked in the interviews were mostly without model answers as follows. “What other things do you think the blocks can do?” “Do you have any other games that can be played with those blocks?” “Have you ever noticed any sensors or something like that in your life?” The questions are listed in a questionnaire, which also contains another two questions about the usability of T-Maze and one about the invitation for our next study recruit.

Reasonably, we did not expect children to become computational thinkers after the one-off practices, but we hoped to find some clues that might answer our questions and preferably got some implications for teaching children computational thinking.

### 5.2. Participants and Apparatus

Seven children (aged 8 in average), 5 girls and 2 boys, participated in this study. They all had some experience in computer use, but none of them had ever known about programming or used any programming tools.

The apparatus was a set of the latest version of T-Maze, placed on an only table in a laboratory room. The table with a chair was in the center of the room and we also made a resting area at the right corner beside the door. One of the two cameras was set up in front left of the table while the other one was behind the table to the left. Each time one child went to the table to complete the tasks with his/her back towards others waiting in the resting area so that they would not interrupt the experiment.

### 5.3. Procedure

The study was conducted in four stages: demonstration, practice, main sessions, and interviews. Before we started, all children watched a demonstration about how to play a simple maze-escape game and create a new maze. We introduced how to use the loop blocks and encouraged children to find the shortest path as they can. Then they were given some minutes to practice freely. After they were ready to start, the main sessions of the study began.

Without time limitation, children could involve in their tasks as much time as they like, but they were asked to give a sign when they were about to begin or to finish and we noted the time for records. In maze-escape session, children were explained to complete a simple escape task first and a complex one afterward. Next in maze creation session, children were told to freely create any maze as they liked. When they finished the creation, we encouraged them to introduce their mazes to others hoping to invite some creative talking. After the main sessions, we conducted one-on-one semi-structured interviews with children and we taped all the conversation for later analysis.

We collected qualitative data in the form of observation notes, photographs, and videotape, from which some quantitative data was also derived. We also collected children's work (the mazes they created). Three researchers were involved in this study: two collected data, while the third acted in the role of lead interpreter.

### 5.4. Results and Discussion

We comprehensively analyzed the observation notes, tapes and interview records, and found answers to the research questions from the analysis of children's words and behaviors, as well as the statistical analysis.

For the first question (easy to use), we were interested in whether children could create their own computer programs. Based on observation notes and an analysis of videotape, we found that children were able to manipulate the tangible blocks to form their own programs. We allowed children to take as much time as they wanted to complete a task. However, we were lucky to see that they neither seemed to take their time nor to rush through the experiment, which gave us the reasonable and valid data in Table 1. Children were able to accomplish both the simple and complex maze-escape tasks within similar period of time. Also, they were even more engaged in maze creation session and spent more time creating and exploring. All children finished the two sessions with their freely-created mazes, some of which had interesting shapes or complex structure with sensor blocks used.

In interviews, when asked whether the tool was easy to use, 2 children chose “very easy”, 4 chose “easy” and one chose “normal”.

For the second research question (interesting to play with), according to the records and videotape, children always showed their interests in sensors. 3 children asked questions about the sensors, like “what are these used for?”, “what if I pressed it longer?”, “only three of them?” Moreover, children were very excited in maze creation section and showed the richest behaviors. For example, one boy said: “I'm going to make the most difficult maze that none of them can get through!” and another boy asked “what if the blocks are not enough to create my maze?” As part of the maze creation session, we asked children not only to demonstrate their mazes, but also to show others the blocks they used to program it. In many cases, children gathered together to the table, just stood by watching and praised for others. In other cases, however, children asked for a competition to prove their own.

In interviews, when asked whether the system was fun, all children confirmed their enjoyment with T-Maze and expressed the wish to participate again in the future. 4 of them voted for the maze creation session to be their favorite.

For the last question (CT concepts in programming), we found some cue that T-Maze has the potential to raise children's awareness about CT and help them understand CT to some extent.

*(i) Abstraction*. The maze metaphor used in T-Maze prompted children to relate their experience about real-world maze to the virtual maze on the screen. They needed the process of abstraction to narrow the escape problem down to something that could be implemented on the computer using T-Maze, as well as mapping the physical blocks to the virtual squares in maze map on screen. Restrictions imposed by the programming environment include an upper bound on the number of blocks and a limit on the size of the programming area (40 cm width by 30 cm height). Abstraction also took place as children designed avatars to react to a limited set of conditions that may be encountered in the real-world maze, such as some roadblocks represented by sensors.

The fact that children all accomplished the tasks successfully was inspiring and we indeed found something related from the observation. In maze-escape session, when the avatar came across a temperature sensor square in the maze, we found 3 children attempting to heat up the physical sensors with their breath. Later in the interviews, we asked why did they do that and one said “Er, my mum always warm my hand with her breath in winter.” Other two said that they always knew their breath was warmer than their hands. The same situation happened with light sensor, where 4 children glanced at the gap between their hands and the light sensor they were covering and muttered something about the light or the avatar on screen. One girl said “it is dark now, go quickly!” and another boy asked to himself that “is it dark enough?” The interesting thing was that one boy bended his body upon the sensor to block the light because he thought it would be quicker to see the outcome. Another remarkable domain where abstraction takes place is maze design and creation. Because the “what you see is what you get” creation process, children could then test complex abstractions quickly and precisely. For example, to create a new maze, the child designed a map, where each square could be represented by certain programming blocks. Then he/she selected only those appropriate for the virtual world in his/her mind.

*(ii) Automation*. Automation occurs as the system executes children's programs. The “program” itself automated “stepping through” and updated virtual avatar's location and direction (representing the veer) at each step. As to this term, we did not expect too much; actually, it was an attempt to find whether children could realize the automatic process. And we indeed found some cue through the study. For example, before the execution, one boy said “listen to my instructions and go!” Another girl jumped for joy when she saw the animation of her avatar traversing through the maze. Besides, we saw 5 children stood up from their seats when the execution began and kept standing by waiting for the successful moment.

In interviews, 4 children expressed their enjoyment while watching the virtual avatar traversing through the maze automatically under their instructions. This was also backed up in the videotape, from which we observed that 3 of them clapped out of joy and cheered on their avatars while watching the animation.

*(iii) Problem Decomposition and Analysis*. T-Maze provides a platform that enables live programming by allowing the code to be adjusted in real time. As the session proceeded, we moved beyond programs consisting of simple sequences of actions and introduced more advanced concepts such as loops and numeric parameter values. Through these activities, we found evidence that children could engage these concepts, reasoning about possible outcomes of different blocks to build, and test every piece of solution until they finally solved the maze-escape problem. Children also performed analysis when they decided whether or not the avatar behaved as expected. If the avatar was stuck or “misbehaved,” it may either mean that their implementation of their control idea is faulty or that some blocks were misplaced.

Mostly, the process of analysis happened implicitly, but we could still find some track from the observation. For example, one child in his complex maze-escape mission determined that he needed five forward blocks to reach the first turning. After learning the loop syntax, he realized that the largest value of a numeric block is five and the loop is limited to 5 times the most, so he needed two more forward blocks. The child then built a program snippet as

 (Start Loop + 5 + Forward + End Loop) ⟶Forward ⟶Forward

In maze creation session, we found 4 children chose to locate the entrance and exit point of a maze at first and then tried to link them through, while others just placed the blocks as they wish and were happy-go-lucky with any shape of mazes. Besides, we observed a sense of “habit” in children's programming that 5 children would add several blocks into the sequence once when it was a simple straight line, while attempted one step at a time in a complex situation (a corner, a sensor square, or a cross), when they would adjust current placement and decide what to do next while observing the feedbacks.

**Figure 7:**
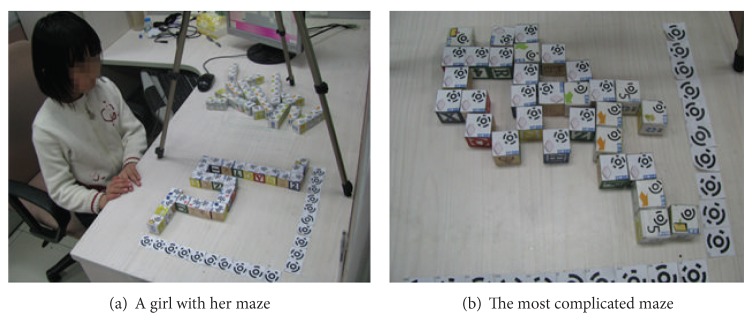
Two mazes collected from maze creation session: a girl with her maze (a) and the complicated maze created by a boy (b).

*(iv) Creativity*. To create a maze, programming became a medium for children's personal and creative expression; in the design of their mazes children engaged their fantasies and built relationships with other pockets of reality that went beyond common thoughts. As we analyzed children's work collected in the study, several interesting mazes surprised us. One girl created her maze shaping like a snake (Figure 7(a)), but she described it as the Great Wall coming out from her textbook. And the boy who claimed to make the most difficult maze used 31 blocks constructing the most complex fish-shape maze (Figure 7(b)). During the creating, he put aside the end block until he finished all the paths, then counted the number of cells in each path trying to find the longest one, where the end block was appended.

Generally, the mazes children created are different from what we designed for T-Maze intrinsically and the descriptions children gave in interviews were also unexpected. For example, one boy created a maze with only one straight line, while he described it as an airport runway. And he talked about his experience with his father at the airport last week right off the reel.

### 5.5. Summary

Though the study is relatively small scale, we can tell from the results that T-Maze has some computational concepts in its programming activities, and children indeed handle those experiences well. This study suggests farther that T-Maze is easy to use for children and they are able to build their own programs in playing and creating activities with T-Maze. The emphasis is that T-Maze has the potential to help raise children's awareness about CT concepts.

In interviews, we also wanted to get some inspirations from children with questions like “what other things do you think the blocks can do?” or “do you think of any other games that can be played with those blocks?” Two children said they would like to control a flying plane with the blocks and 3 others wished to help their mothers with some household duties like cooking, cleaning, or furnishing their rooms. Their unlimited imagination inspired us in the revision and upgrade of T-Maze in the future.

## 6. Conclusion and Future Work

T-Maze is designed for children aged 5–9 to help cultivate their computational thinking skills. It allows children to play maze-escape games and create their own mazes by building computer programs out of wooden blocks. We conducted a user study to verify its ease of use and the potential capability to help children with computational thinking. The results showed that T-Maze has the promising potential to help children cultivate some computational thinking awareness like abstraction, automation, problem decomposition, and analysis.

In the future, we will develop more games and scenarios, design more kinds of programming blocks, and enrich the types of sensors. More and further user studies are needed to improve T-Maze for better user experience and skills nurturing capability. Besides, as an upgrade, we may provide a collaborative tangible programming tool for children to experience the fun of cooperation.

## Figures and Tables

**Table 1:** The time (unit: minute) and blocks children used in the study.

Number	Maze escape session				Maze creation session	
	Simple		Complex			
	Time (min)	Blocks	Time (min)	Blocks	Time (min)	Blocks
1	3.50	9.00	7.00	18.00	9.00	18.00
2	3.00	9.00	5.00	19.00	10.00	26.00
3	4.50	9.00	7.00	21.00	13.00	18.00
4	4.50	9.00	9.00	22.00	8.00	16.00
5	3.00	9.00	8.00	20.00	11.00	31.00
6	4.00	8.00	9.00	21.00	10.00	26.00
7	4.00	9.00	8.00	18.00	9.00	25.00

Avg.	3.78	8.86	7.57	19.86	9.86	22.86
